# Supplementary figures and images for: Biased placement of Mitochondria fission facilitates asymmetric inheritance of protein aggregates during yeast cell division
Source: PLoS Comput Biol. 2023 Nov 27;19(11):e1011588. doi: 10.1371/journal.pcbi.1011588 (PMC10703421; doi:10.1371/journal.pcbi.1011588)

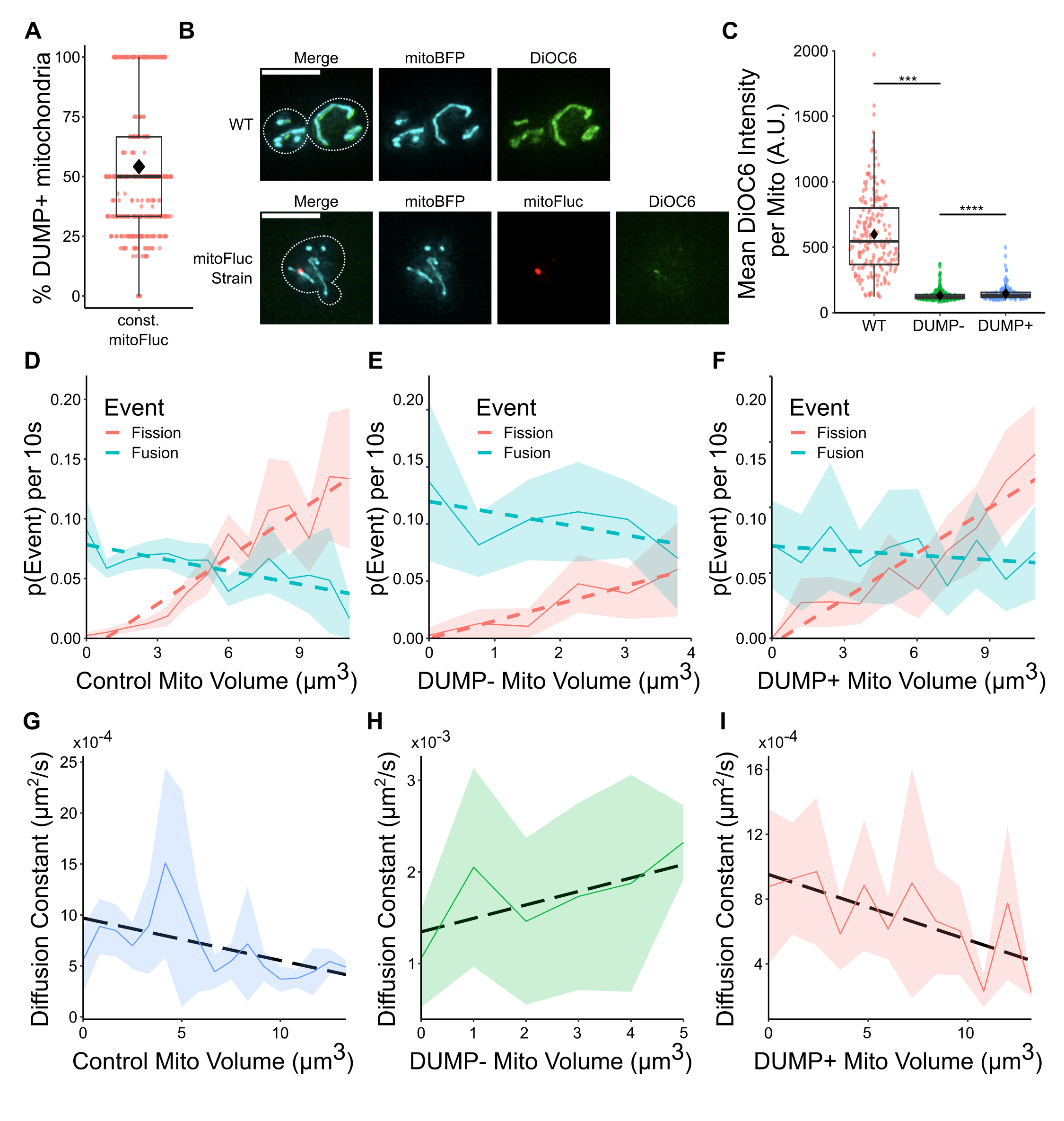

Supplement: S1 Fig — (A) Proportion of DUMP+ mitochondria of all mitochondria in mitoFluc cells, n = 413 cells (B) Representative snapshots of DiOC6 stained cells. Top row: WT cells, bottom row: Cells expressing mitoFluc constitutively. (C) Mean DiOC6 fluorescence intensity per mitochondria. n = 206, 263, 141 for WT, DUMP- and DUMP+ mitochondria respectively from 39 WT, 74 mitoFluc cells (WT = mitoBFP only). Mann-Whitney U test, ****p<0.0001. (D-F) Bootstrapped probability of fission and fusion per 10s, stratified by mitochondria volume. Shaded ribbons represent 95% of data range (from 0.025 to 0.975 quantile), with line indicating mean. Dashed bold line indicates linear fit. Red = p(Fission) per 10s, blue = p(Fusion) per 10s. (D, G) WT mitochondria = Tom70-GFP only strain. (D) WT mitochondria p(Event) by volume; n = 208 cell movies represented; mitochondria per bin n≥68. (E) DUMP- mitochondria p(Event) by volume; n = 134 cell movies; mitochondria per bin n≥59. (F) DUMP+ mitochondria p(Event) by volume; n = 134 cell movies; mitochondria per bin n≥57. (G-I) Diffusion constant by mitochondria volume. Dashed black line indicates linear fit. (G) Control mitochondria diffusivity by volume; n = 184 cell movies. (H) DUMP- mitochondria diffusivity by volume; n = 106 cell movies. (I) DUMP+ mitochondria diffusivity by volume; n = 119 cell movies. (D-I) Linear fit coefficients are in Table 1. (TIF) [file pcbi.1011588.s001.tif]

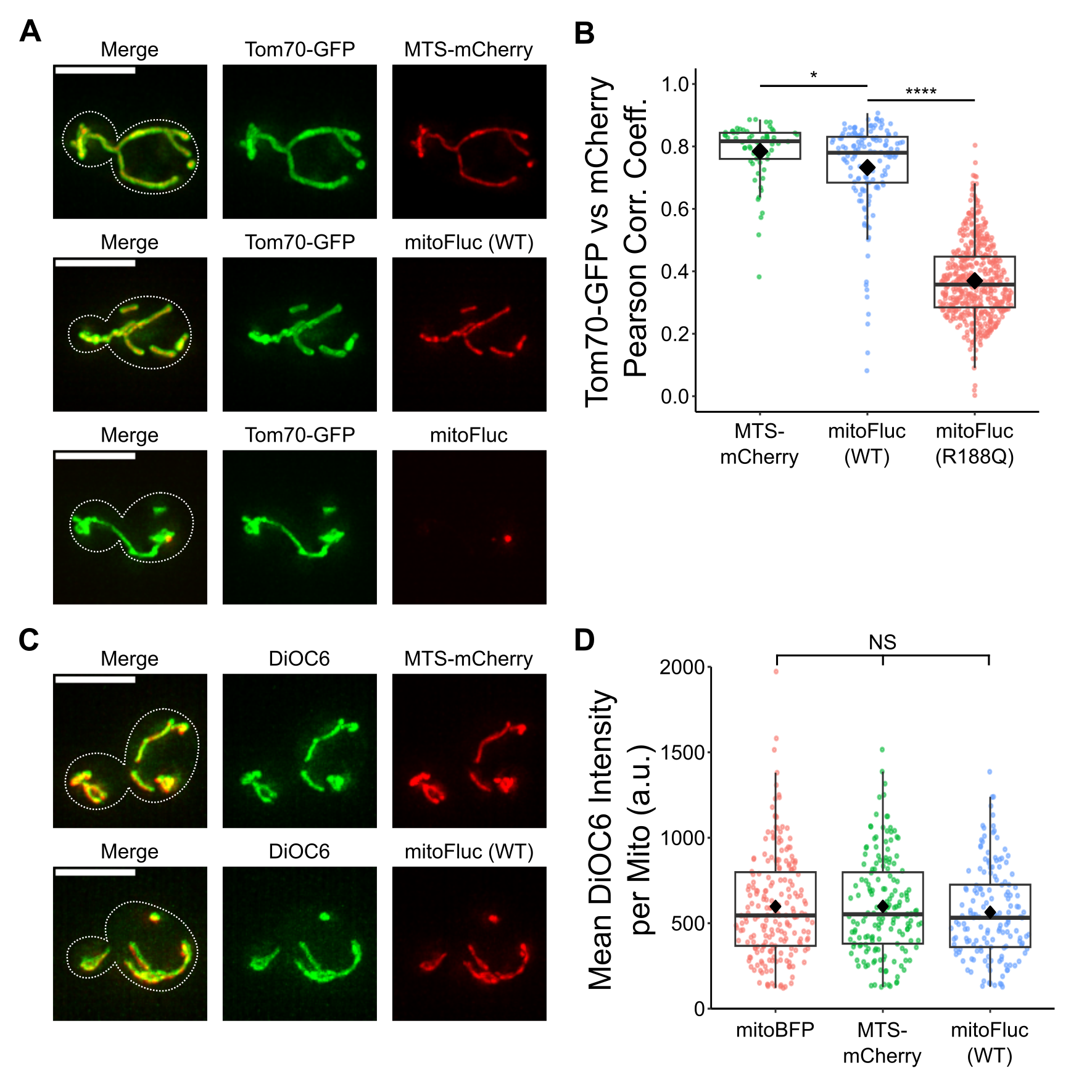

Supplement: S2 Fig — (A, C) Scale bar = 5μm shown in merged images. White dashed line demarcates cell boundaries traced from DIC images. (A) Representative images of cells expressing Tom70-GFP (as a mitochondria marker) with MTS-mCherry, mitoFluc (WT), vs. mitoFluc. mitoFluc(WT) contains a non-mutant version of luciferase that is not prone to misfolding. (B) Pearson correlation coefficient of Tom70-GFP signal v.s. mCherry signal for colocalization analysis of mCherry signal with respect to the mitochondria structure. N = 62, 139, 503 for MTS-mCherry, mitoFluc (WT), and mitoFluc (R188Q) respectively. *p<0.05, ***p<0.001, Wilcoxon rank sum test. (C) Representative snapshots of DiOC6 stained cells. Top row: Cells expressing MTS-mCherry constitutively, bottom row: Cells expressing mitoFluc(WT) constitutively. (D) Mean DiOC6 fluorescence intensity per mitochondria. n = 206, 165, 155 for mitoBFP, MTS-mCherry and mitoFluc(WT) mitochondria respectively from 39 mitoBFP cells, 35 MTS-mCherry cells, and 37 mitoFluc(WT) cells. Mann-Whitney U test, NS>0.9 for all comparisons. (TIF) [file pcbi.1011588.s002.tif]

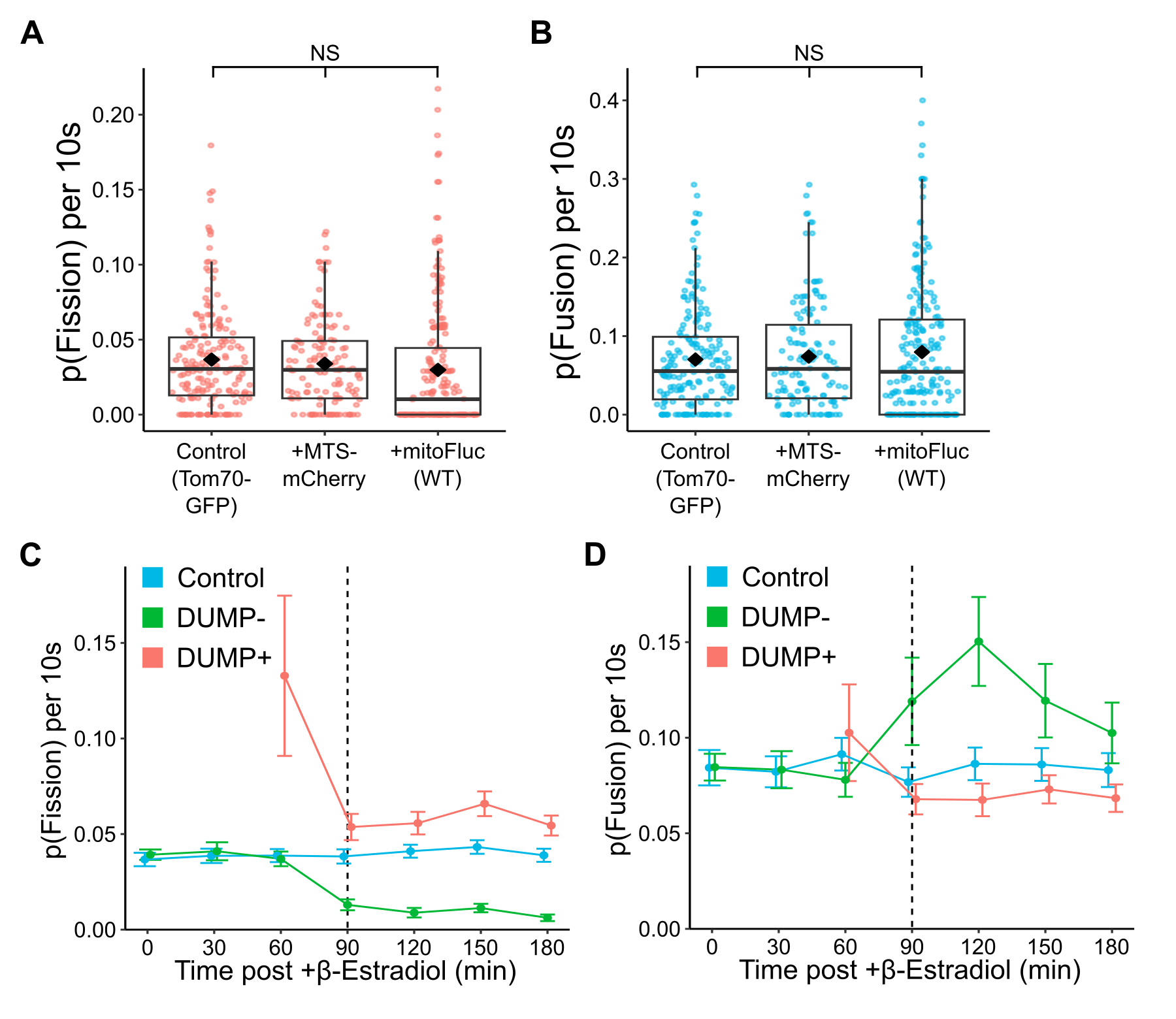

Supplement: S3 Fig — Probability of mitochondria to undergo fission (A), fusion (B) per 10s by mitochondria type. Each point represents a single cell’s population of the associated mitochondria type. N = 184, 98, 119 single cell movies for Control (Tom70-GFP only), Control + MTS-mCherry, Control + mitoFluc(WT) respectively. Tukey’s HSD multiple comparisons test. (A) NS = 0.961 for Control vs. MTS-mCherry, 0.968 Control vs. mitoFluc(WT), and 0.872 MTS-mCherry vs. mitoFluc(WT). (B) NS = 0.998 for Control vs. MTS-mCherry, 0.297 Control vs. mitoFluc(WT), and 0.399 MTS-mCherry vs. mitoFluc(WT). Probability of mitochondria to undergo fission (C), fusion (D) per 10s by mitochondria type over time after induced expression of mitoFluc. N≥57 cell movies for all timestamps for induced mitoFluc strain, 121 for Control (EtOH). Black dashed line marks when DUMPs are visible under microscope in most budding cells. Mean±SEM shown. (TIF) [file pcbi.1011588.s003.tif]

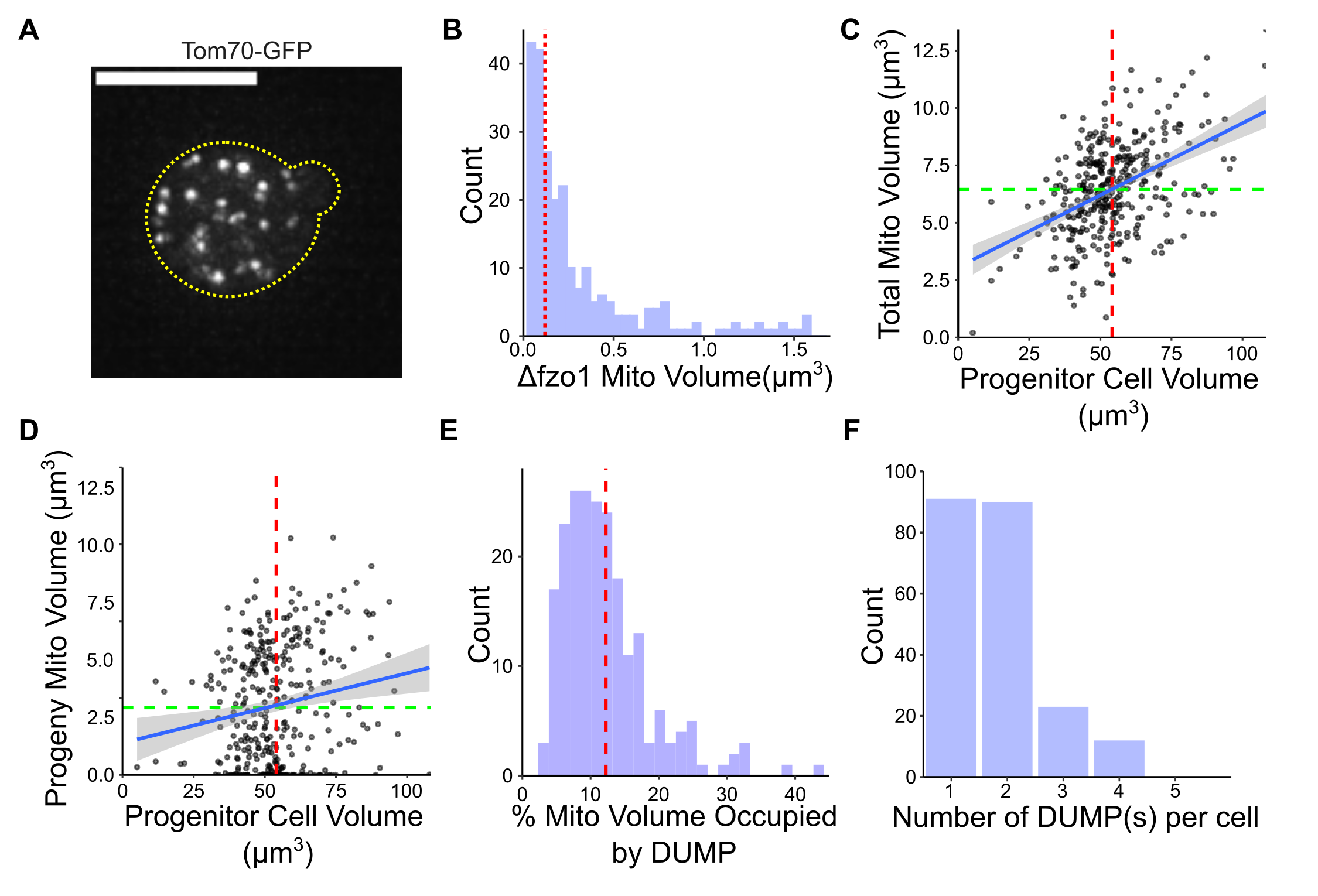

Supplement: S4 Fig — (A) Representative snapshot of cell with Tom70-GFP, Δfzo1. Yellow dashed line demarcates cell boundaries traced from DIC images. (B) Mitochondria volume distribution for Δfzo1 mutant. Red vertical line marks mean = 0.12μm3. n = 141 cells represented. (C-D) Linear regression (blue line) with 95% confidence interval (shaded). Red dashed line indicates mean mother cell volume (54.0μm3) used in simulation. n = 346 cells represented. (C) Mother cell volume vs total mitochondria volume in mother and bud. Green dashed line indicates total mitochondria volume (6.56μm3) used for simulation. (D) Mother cell volume vs. mitochondria volume in bud. Green dashed line indicates mitochondria volume (1.83μm3) used for simulation in inheritance. (E) Distribution of percentage of total mitochondria volume occupied by DUMP. Mean = 11.2% (red-dashed line). (F) Number of DUMPs observed in each cell with constitutive mitoFluc expression, n = 216 cells. (TIF) [file pcbi.1011588.s004.tif]

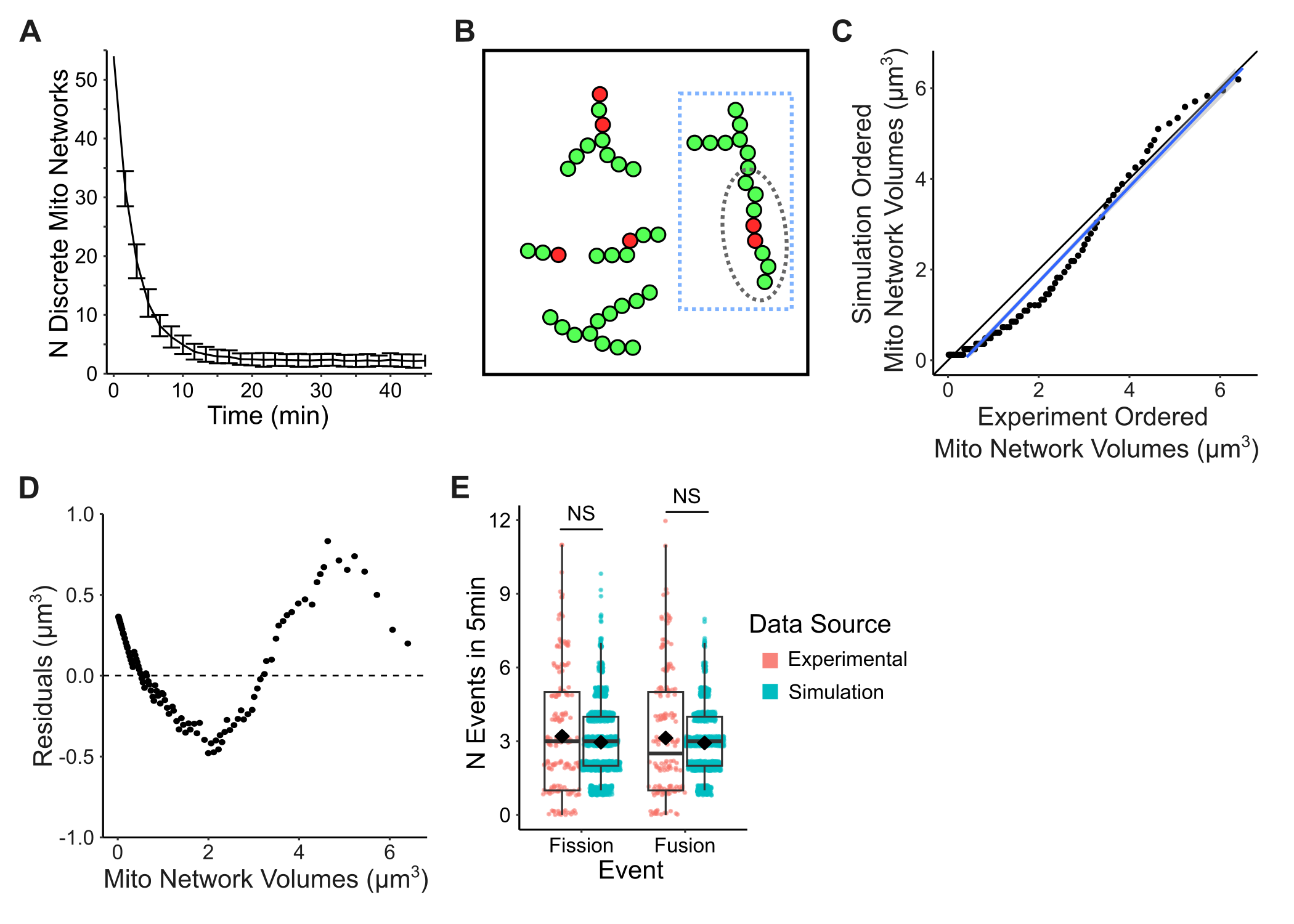

Supplement: S5 Fig — (A) Number of discrete mitochondria networks over time. Simulations ran under all WT parameters for motion, probability of fission/fusion, and no targeted fission (n = 100). (B) Two-step algorithm for inheritance implemented in the model. Step (1): largest mitochondria subnetwork by volume is determined (blue dotted box); Step (2): the tip-most N particles (grey dashed ellipse, N = 8 inherited particles for this schematic, for simulation it is set to Ninherit, Table 1) from the largest branch from the subnetwork is selected as inherited by the bud. Figure is simplified for clarity. (C) Quantile-quantile plot of experimentally measured vs. simulated mitochondria network volume distributions. WT strain (Tom70-GFP) was used as experimental data, simulation data was acquired from running with WT parameters. Volumes are ordered from smallest to largest, with black line marking if both distributions were identical. Blue line represents linear fit to data. N = 1087, 4516 mitochondria for experimental and simulation data, with 346, 1212 cells represented respectively. Slope = 0.977±0.019, R2 = 0.961. (D) Residuals from QQ plot in (C); mean absolute residuals = 0.283 μm3. (E) Number of fission or fusion events that occurred within a 5-minute window for experimental (n = 184) vs simulation (n = 1212) data. Mann Whitney U-test NS p = 0.25 for fusion, p = 0.9294 for fission. (TIF) [file pcbi.1011588.s005.tif]

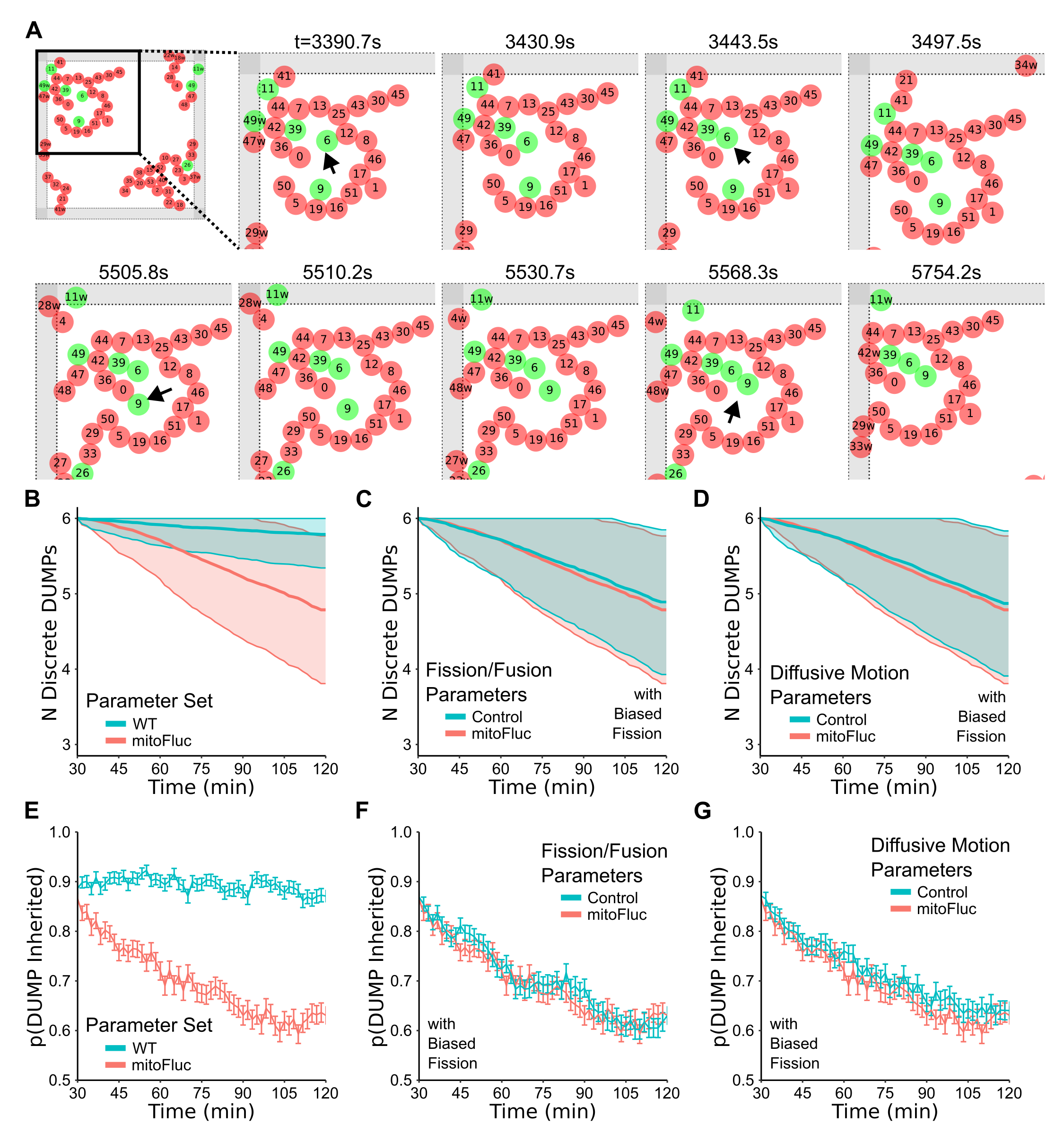

Supplement: S6 Fig — (A) Representative model simulation illustrating that biased fission facilitates clustering of DUMPs. Specifically, biased fission prevents permanent attachment of DUMPs to non-DUMP regions of DUMP+ mitochondria. Time is shown above each box, with black arrows indicating targeted fission followed by DUMP clustering. Green = DUMP+, red = DUMP- mitochondria. Particle numbers are for tracking only; grey box indicates periodic boundary condition, with “w” marked on particles that have wrapped around. (B) Number of DUMP(s) in simulation over time with associated (E) bootstrapped probability of DUMP inheritance over time with WT vs mitoFluc parameters (Table 2). (B, E) n = 643, 644 simulations respectively. (C) Number of DUMP(s) in simulation over time with associated (F) bootstrapped probability of DUMP inheritance over time with control (KfisWT,KfusWT) vs mitoFluc (KfisDUMP+/−,KfusDUMP+/−) fission/fusion. (C, F) n = 645, 644 simulations respectively, biased fission is on, with DDUMP+/− fixed. (C) Number of DUMP(s) in simulation over time with associated (F) bootstrapped probability of DUMP inheritance over time with control (DWT) vs. mitoFluc (DDUMP+/−) diffusive motion. (C, F) n = 645, 645 simulations respectively, biased fission is on, with KfisDUMP+/−,KfusDUMP+/− fixed. (B-D) Mean±SEM, (E-G) Mean±SD (standard deviation) are shown. (TIF) [file pcbi.1011588.s006.tif]

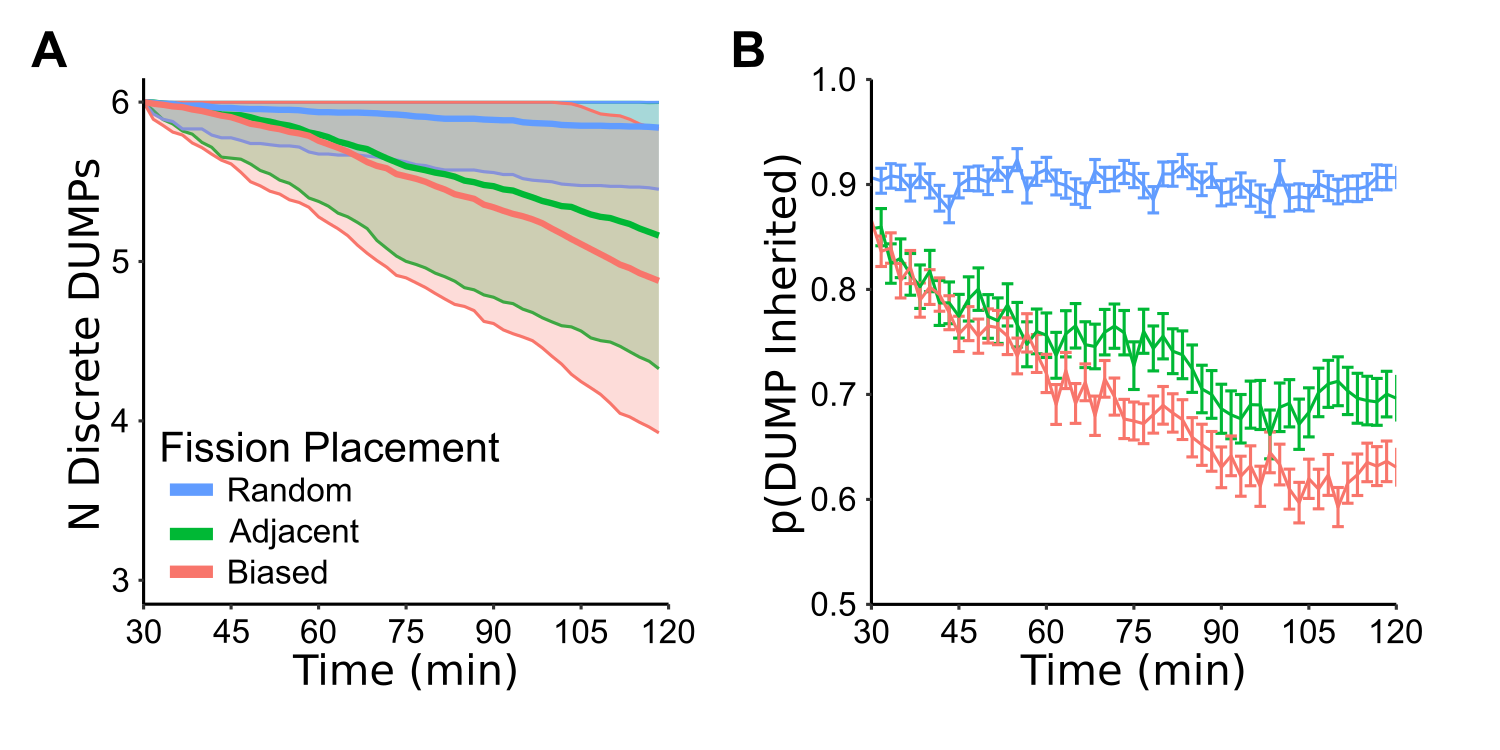

Supplement: S7 Fig — (A) Number of DUMP(s) in simulation over time with associated (B) bootstrapped probability of DUMP inheritance over time with mitoFluc parameters with different fission placement conditions with mitoFluc parameters (Table 2). Legend for (B) is identical to in (A). Different fission placement conditions are random, adjacent, and biased as described under Methods, “Simulation Routine”. (A, B) N = 643, 412, 644 simulations for random, adjacent, and biased fission placement respectively. (A) Mean±SEM, (B) Mean±SD are shown. (TIF) [file pcbi.1011588.s007.tif]

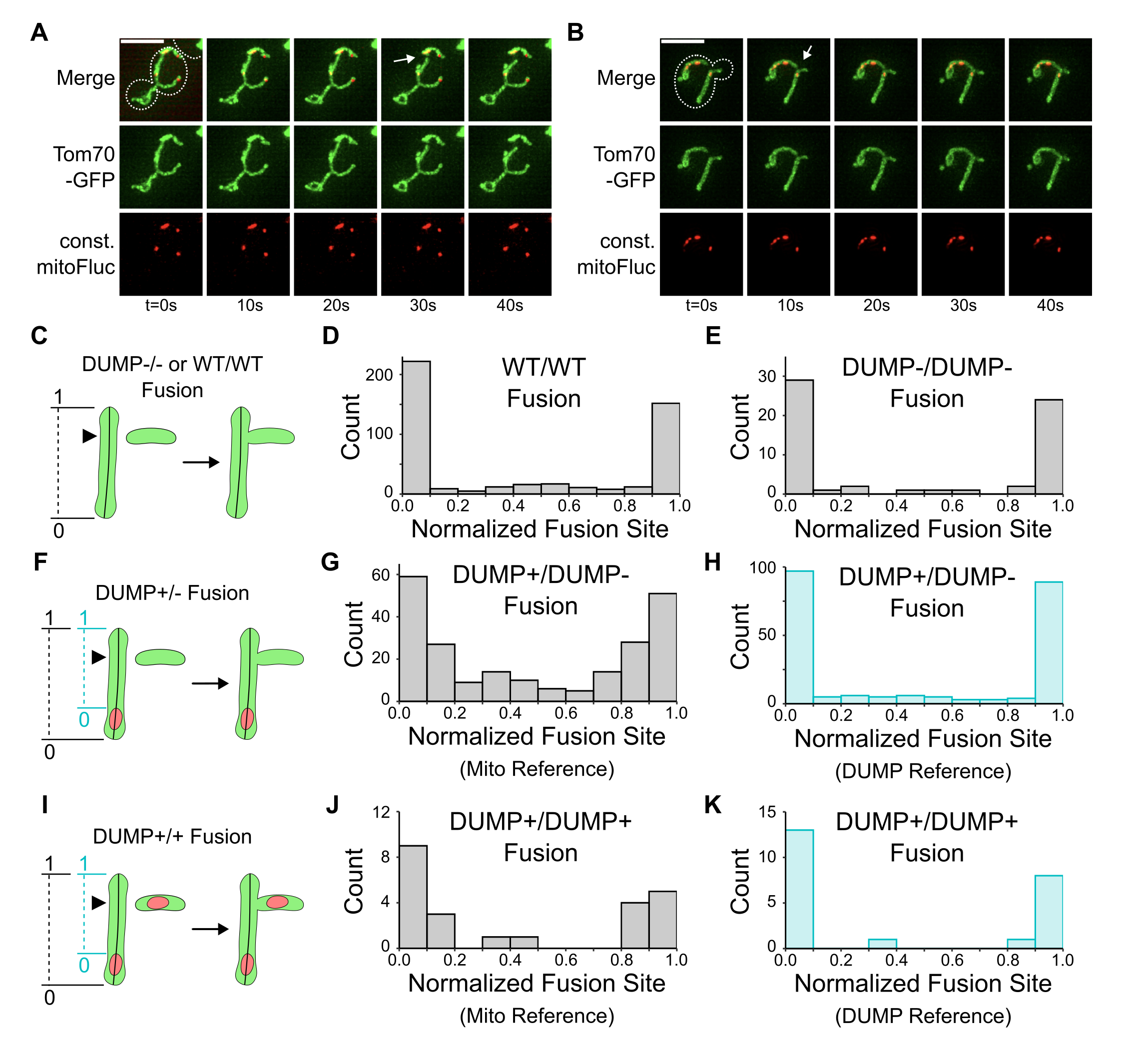

Supplement: S8 Fig — (A) Representative timeseries showing fission occur between two DUMPs. White arrow indicates site of future fission. (B) Representative timeseries showing tip-to-tip fusion occur between two mitochondria in mitoFluc cells. White arrow indicates site of future fusion. (C, F, I) Future fusion site is marked by black triangle, with dotted projection indicating the length of mitochondria measured. Mitochondria skeleton marked by black solid line spanning green mitochondria, DUMP marked red.(C) Fusion bias normalization procedure for between mitochondria undergoing fusion in WT cells, or between DUMP- mitochondria in mitoFluc cells. (D) Normalized fusion placement in WT cells. n = 147 cells, 464 fusion events observed. (E) Normalized fusion placement in mitoFluc cells, between DUMP- and DUMP- mitochondria. n = 23 cells, 61 fusion events observed. (F, I) Black dotted projection is with respect to the whole mitochondria backbone length (with backbone end closest to DUMP set to 0), while blue dotted projection is with respect to the DUMP boundary (set to 0). (F) Fusion bias normalization procedure for between DUMP+ and DUMP- mitochondria in mitoFluc cells. Color is matched to (G, H). n = 77 cells, 223 fusion events observed. Normalized fusion placement in mitoFluc cells between DUMP+ and DUMP- mitochondria normalized to mitochondria backbone (G), and with respect to DUMP location (H). (G, H) Pearson correlation = 0.974 (I) Fusion bias normalization procedure for between DUMP+ and DUMP+ mitochondria in mitoFluc cells. Color is matched to (J, K). n = 15 cells, 23 fusion events observed. Normalized fusion placement in mitoFluc cells between DUMP+ and DUMP+ mitochondria normalized to mitochondria backbone(J), and with respect to DUMP location (K) Normalized fusion placement in mitoFluc cells between DUMP+ and DUMP+ mitochondria. (J, K) Pearson correlation = 0.976. (TIF) [file pcbi.1011588.s008.tif]

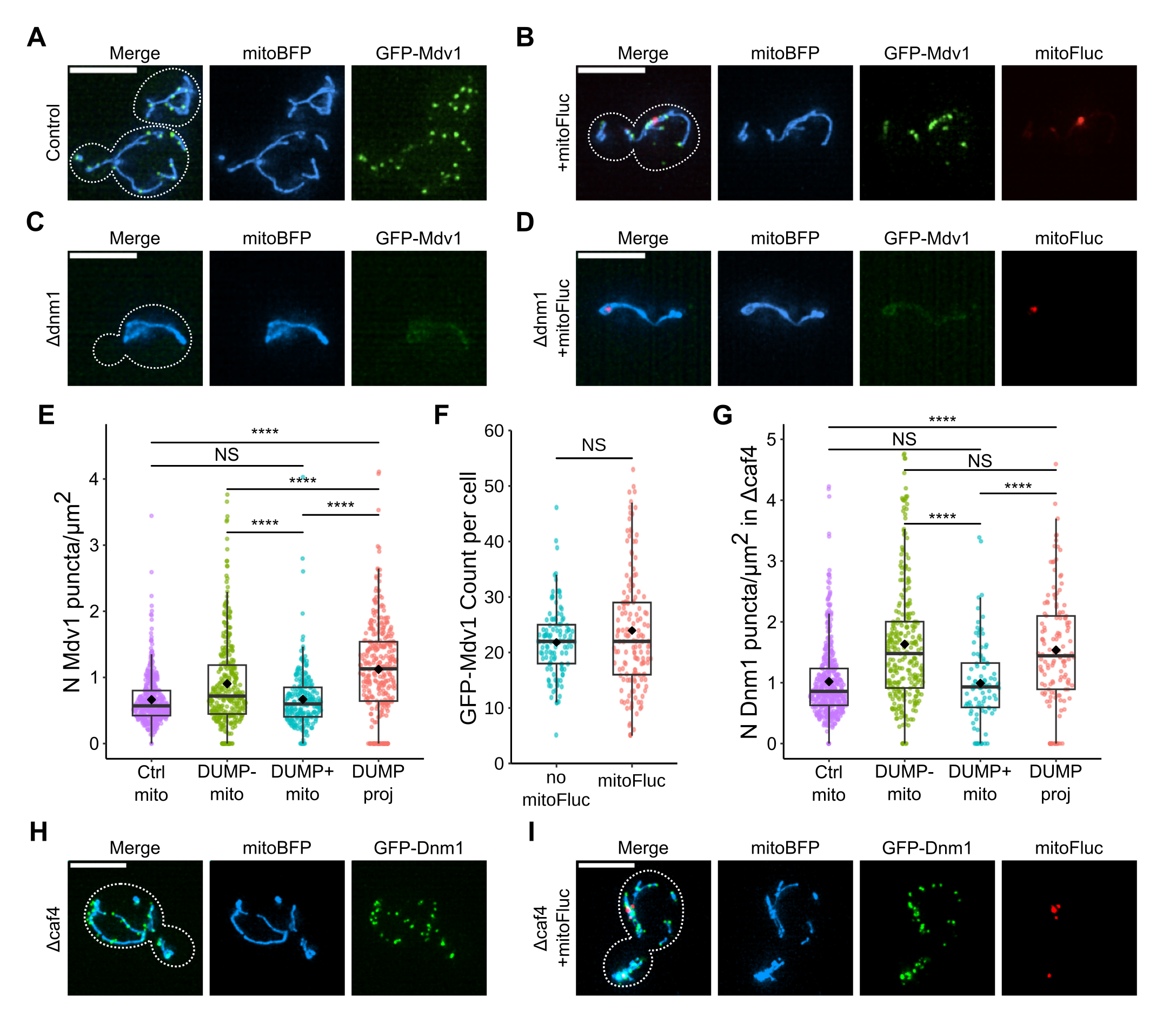

Supplement: S9 Fig — Representative GFP-Mdv1 localization in non-mitoFluc (A) vs mitoFluc cells (B). Representative GFP-Mdv1 localization in Δdnm1 cells without (C) and with mitoFluc (D). (E) Mdv1 puncta surface density. Quantification scheme identical to Fig 3A. Control = mitoBFP, GFP-Mdv1 cells; n = 119 cells with 469 mitochondria total. n = 159 cells for mitoFluc, with 583 mitochondria total, 337 which were DUMP-, 246 DUMP+; n = 335 DUMPs were found in DUMP+ mitochondria, which were used to evaluate DUMP projection surface densities. Mann Whitney U test, NS p = 0.948; ***p<0.001; ****p<0.0001. (F) Total number of GFP-Mdv1 puncta per cell with no-mitoFluc vs constitutive mitoFluc expression, n = 119, 159 cells respectively. Mann Whitney U-test, NS p = 0.221. (G) Dnm1 puncta surface density in Δcaf4 cells. Quantification scheme identical to Fig 5E. Control = mitoBFP, GFP-Dnm1, Δcaf4 cells; n = 72 cells with 494 mitochondria total. n = 58 cells with mitoFluc, with 406 mitochondria total, 299 which were DUMP-, 107 DUMP+; n = 142 DUMPs were found in DUMP+ mitochondria, which were used to evaluate DUMP projection surface densities. Mann Whitney U test, NS p = 0.996; ****p<0.0001. (H) Representative GFP-Dnm1 localization in Δcaf4 cells. (I) Representative GFP-Dnm1 localization in mitoFluc, Δcaf4 cells. All images in the GFP channel are displayed with consistent intensity range, brightness, and contrast settings for accurate comparison. (TIF) [file pcbi.1011588.s009.tif]

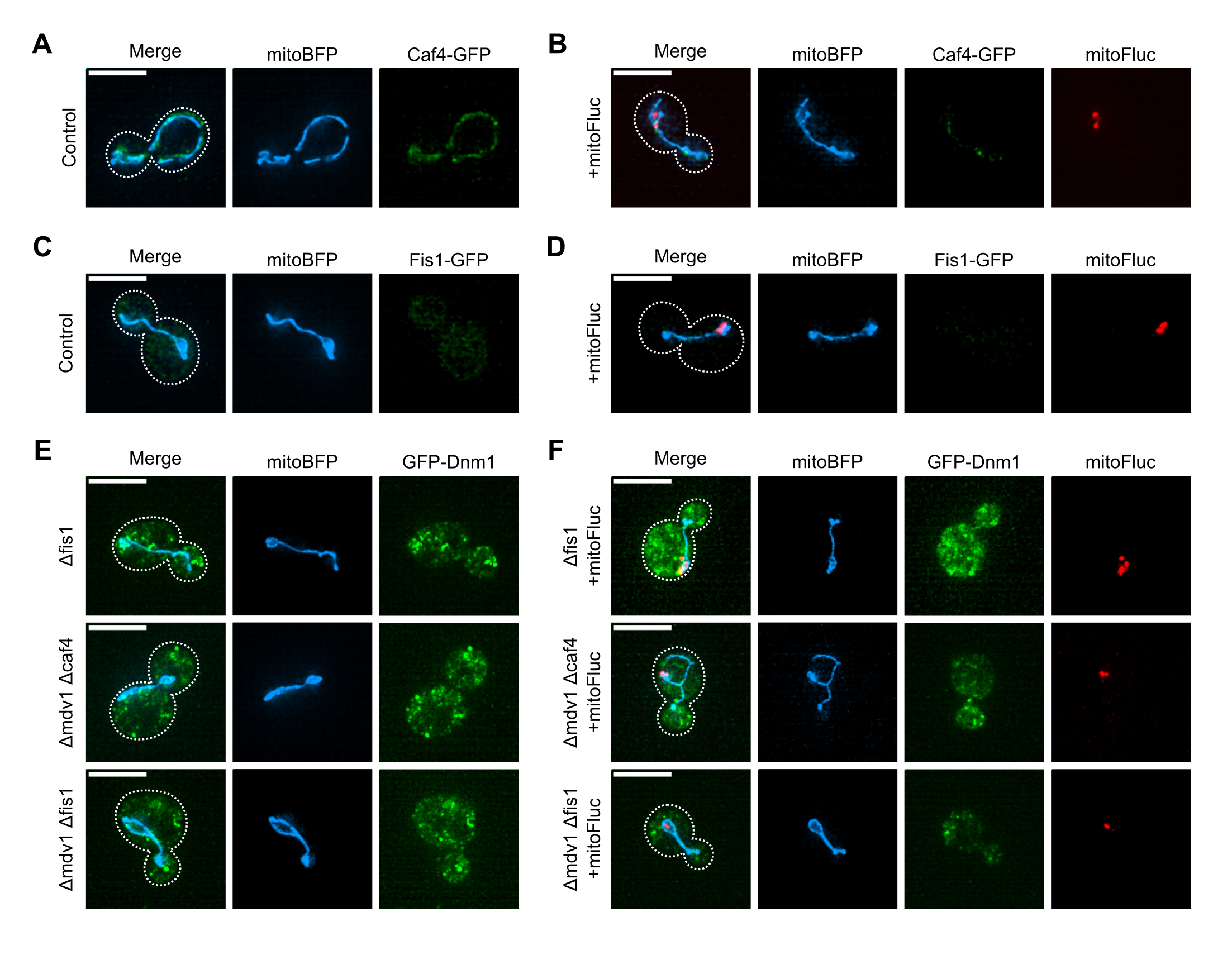

Supplement: S10 Fig — Representative Caf4-GFP localization in control (A) vs mitoFluc cells (B). Representative Fis1-GFP localization in control (C) vs mitoFluc cells (D). Representative GFP-Dnm1 localization in Δfis1, Δmdv1Δcaf4, Δmdv1Δfis1, without (E) and with (F) mitoFluc. All images in the GFP channel are displayed with consistent intensity range, brightness, and contrast settings for accurate comparison. (TIF) [file pcbi.1011588.s010.tif]

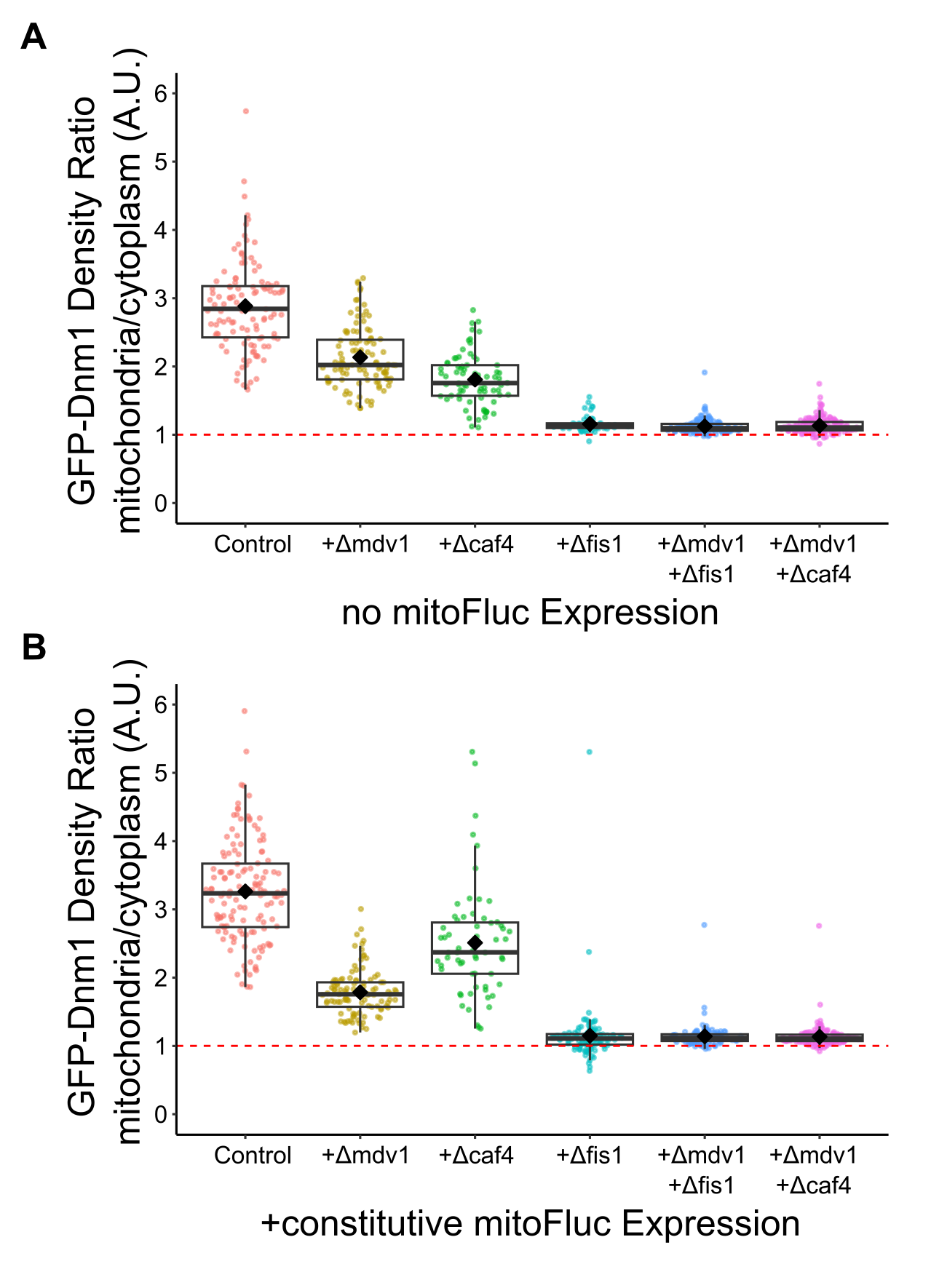

Supplement: S11 Fig — Ratio of GFP-Dnm1 density on mitochondria over cytoplasm when cells do not have constitutive expression of mitoFluc (A) vs. when mitoFluc is constitutively expressed (B). Horizontal red-dashed line marks a ratio of 1.0, which would indicate that the fluorescence density of GFP-Dnm1 is identical on the mitochondria and cytosol per μm3. (A, B) All labels on X axis with “+” indicate additional mutations applied to control strain. (A) Control = mitoBFP, GFP-Dnm1. N = 114, 103, 52, 72, 126, 145 cells for Control, +Δmdv1, +Δcaf4, +Δfis1, +Δmdv1Δfis1, +Δmdv1Δcaf4 respectively. (B) Control = mitoBFP, GFP-Dnm1, mitoFluc. N = 136, 102, 109, 63, 111, 141 cells for Control, +Δmdv1, +Δcaf4, +Δfis1, +Δmdv1Δfis1, +Δmdv1Δcaf4 respectively. Black diamond marks mean. (TIF) [file pcbi.1011588.s011.tif]

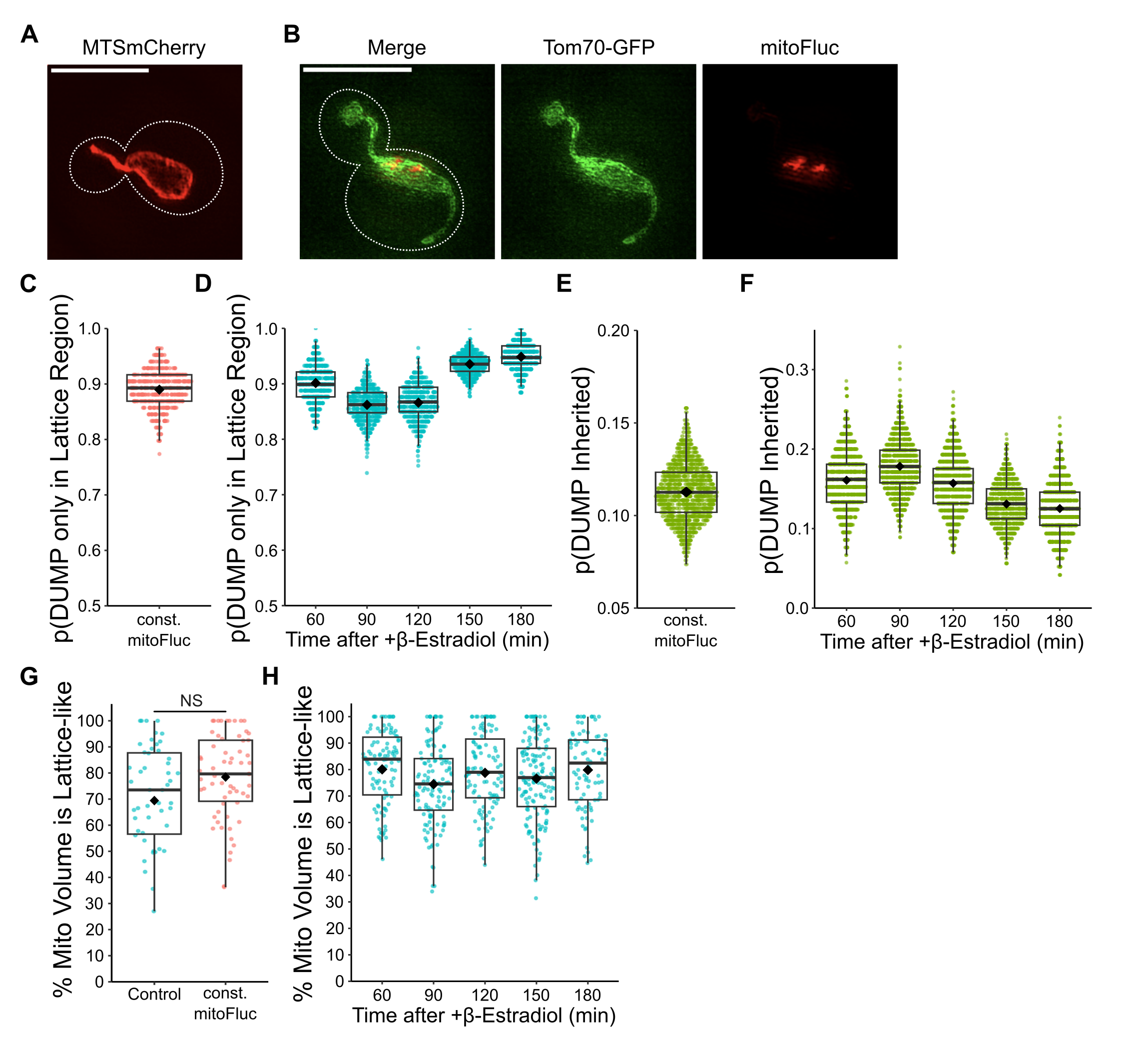

Supplement: S12 Fig — (A-B) Scale bar = 5μm shown in merged images. White dashed line demarcates cell boundaries traced from DIC images. (A) Representative structured illumination microscopy image of MTSmCherry Δdnm1 cell showing mitochondria net structures, (B) of Tom70-GFP, Δdnm1 with constitutive mitoFluc. (C-F) Bootstrapped probability of observing DUMP in net-like domain of mitochondria. Iterations = 500, sample size = n. (C) Δdnm1 cells with constitutive mitoFluc expression, n = 84 cells. (D) Δdnm1 cells with induced mitoFluc expression, n = 89, 139, 113, 155, 95 for timestamps 60, 90, 120, 150, 180 respectively. Bootstrapped probability of DUMP inheritance in cells expressing mitoFluc constitutively (E) n = 462 cells or induced (F) over time n = 105, 146, 114, 160, 96 for timestamps 60, 90, 120, 150, 180 respectively. Bootstrap sampling from sample population of size n; iterations = 500, sampling size = n. (G) Percentage of total mitochondria volume is lattice-like in Δdnm1 cells. n = 51, 62 cells for control (Δdnm1 only) and Δdnm1 with constitutive mitoFluc, respectively. Two tailed t-test, NS (p = 0.069). (H) Percentage of total mitochondria volume is lattice-like in Δdnm1 cells with inducible mitoFluc after exposure to β-Estradiol. n = 106, 126, 111, 150, 85 for timestamps 60, 90, 120, 150, 180 respectively. (TIF) [file pcbi.1011588.s012.tif]

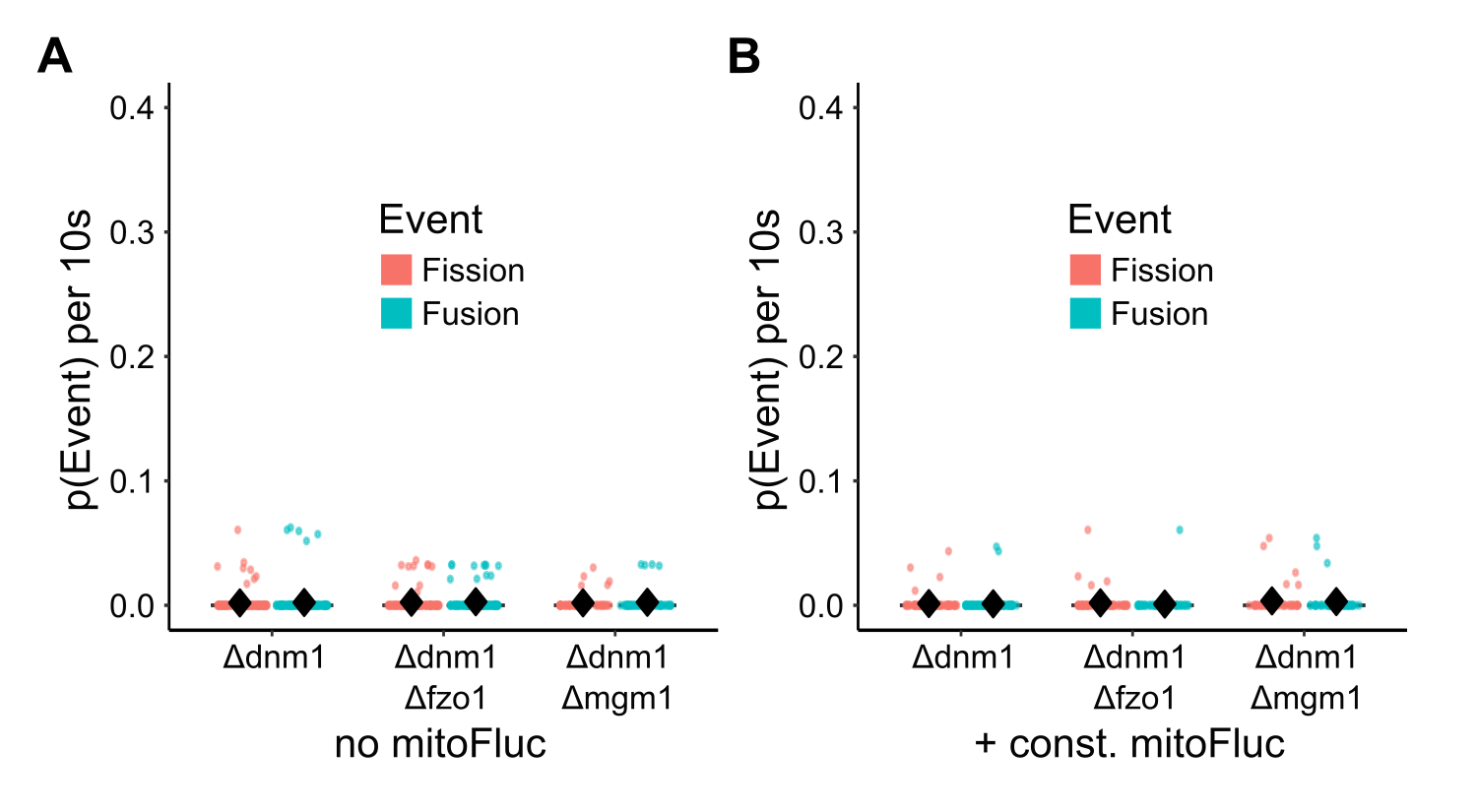

Supplement: S13 Fig — (A, B) Probability of fission and fusion per 10 seconds. (A) With no mitoFluc expression. n = 139, 150, 59 cell movies for Δdnm1, Δdnm1Δfzo1, Δdnm1Δmgm1, respectively. (B) With constitutive mitoFluc expression. n = 89, 78, 49 cell movies for Δdnm1, Δdnm1Δfzo1, Δdnm1Δmgm1, respectively. (TIF) [file pcbi.1011588.s013.tif]
