# Supplementary material for: Biased placement of Mitochondria fission facilitates asymmetric inheritance of protein aggregates during yeast cell division
Source: PLoS Comput Biol. 2023 Nov 27;19(11):e1011588. doi: 10.1371/journal.pcbi.1011588 (PMC10703421; doi:10.1371/journal.pcbi.1011588)
Supplement: Attachment — Submitted filename: review_comments_r1.2.1_clean.pdf [file pcbi.1011588.s014.pdf]

Dear Editors:

We thank the reviewers for their generous comments on the manuscript and have edited the manuscript to address their concerns as follows:

Reviewer 1:

R1.1: Because the paper appears to be more heavily weighted toward experiments than computational studies, I wonder if a different journal (e.g. PLoS Biology or MBoC) might be a more appropriate venue for it.

We appreciate the reviewer's perspective on the balance of experimental versus computational content in our manuscript. While our study does emphasize experimental data, the computational studies are integral to our investigation and assist in providing a holistic and quantitative understanding of the underlying mechanisms. The synergy between our experimental and computational approaches, we believe, offers a more comprehensive insight which is in line with the interdisciplinary ethos of PLOS Computational Biology. Papers of similar styles are published in PLOS Computational Biology: *e.g.*, “An Exclusion Zone for Ca2+ Channels around Docked Vesicles Explains Release Control by Multiple Channels at a CNS Synapse” by Keller et al, PLoS Computational Biol 11(5): e1004253. doi:10.1371/journal.pcbi.1004253.

We therefore believe our paper fits well within the scope of the journal, not only due to the methods employed but also the broader implications and relevance of our findings to the journal's readership.

R1.2: In the section “Biased fission is predicted to underlie the asymmetric retention of DUMP via DUMP clustering” the authors state that in their model “When biased fission was present, fission would only occur between adjacent DUMP marked and non-DUMP particles.” However, their experiments seem to indicate fission also occurs between DUMP sites (Figs. 3F and G). How do the authors reconcile this observation with their model?

We apologize for this inaccurate statement. In our model, fission can occur randomly between DUMP sites, indeed based on our observation (Figs. 3F and G). In the statement of “fission would only occur between adjacent DUMP marked and non-DUMP particles”, the fission refers to biased fission. We therefore changed the sentence as “When biased fission was present, it would only occur between adjacent DUMP marked and non-DUMP particles” (line 333). Please see the main text in lines 350-354, 366-368, 951-955

R1.3: The assumption that fission only occurs between DUMP and non-DUMP particles appears to be critical for generating DUMP clusters because when two DUMP particles fuse, they are unlikely to dissociate. This assumption also appears to be necessary for the mitochondria containing the DUMP clusters not to be the longest branch, because when a non-DUMP particle associates with the DUMP cluster, it is likely to undergo fission, keeping the size of this branch short. What happens in the simulations if instead of assuming biased fission only occurs between DUMP and non-DUMP particles, fission is simply increased adjacent to DUMP particles? If this is not sufficient to generate DUMP clusters that are retained in the mother, is there a way to show experimentally that two nearby DUMP sites are less likely to undergo fission then equal length portions of the mitochondrial chain that are not between to DUMP sites?

We note that fission frequently happens near DUMPs (Fig 3I), it is not always immediately adjacent to them (Fig 3C). Nonetheless, setting biased fission not immediately adjacent to DUMP particles still resulted in clustering of DUMPs over time in our model, with also a reduction in probability of inheritance (Fig. S7, lines 958-965).

R1.4: Simulation results for DUMP clustering in the form of time series or movies should be presented, so readers can see what the simulation results look like and compare them with the experimental results.

We appreciate the reviewer's emphasis on visualizing simulation results. To address this, we have included a sample time series in Fig S6A, showing key moments in the simulation where DUMP clusters. Given each simulation has 72,000 frames, producing a full movie is challenging. Instead, we share selected frames that highlight the crucial dynamics, offering a representative view without presenting all frames. Furthermore, for those interested in visualizing a running simulation, we have provided instructions in our code repository for running the simulation with visualization, as well as the analysis code needed to identify specific frames within the simulation where DUMPs cluster (lines 1043-1044).

<u>Reviewer 2:</u>

R2.1: Cells expressed mitochondria-targeted misfolded proteins as a model system. How much overexpression is occurring and can this be controlled? There was no mention or consideration of this when the experiments are introduced or in the methods section.

We employed the GAP promoter for the constitutive expression of mitoFluc and later introduced b-estradiol-induced expression via the GAL1 promoter, as detailed in a previous publication and now further clarified in our methods section (lines 676-688). Both are yeast promoters that are often used to produce high-level expression. Because mitoFluc is a model misfolding-prone protein rather than a native yeast protein, it is not relevant to consider if it is “over-expressed”. We used high-level expression to consistently report DUMP formation for examination by live imaging.

R2.2: Does the expression of a NON-Mutant fly luciferase distribute uniformly throughout the mitos? And does this not impact mitochondrial dynamics/behavior in the same way? This might distinguish the impact of the gene vs the misfolded protein response.

Expression of non-mutant luciferase (MTS-Fluc(WT)-mCherry) results in a uniform distribution throughout the mitochondria. Additional controls that we tested include MTS-mCherry, where

there is no luciferase, which similarly exhibits a uniform distribution throughout the mitochondria. We do not observe any distinct differences in mitochondria fission/fusion rate or membrane potential, as shown in Fig S2, S3.

Furthermore, upon inducing the mutant version, (MTS-FlucSM-mCherry), we notice a deviation in the fission/fusion rates 30 minutes post-induction from the baseline rates observed before induction (Fig S3C, D). Given this data combined with our previous findings, it's evident that the altered mitochondrial dynamics arise specifically due to the misfolded protein forming DUMPs. We have included the dynamics data and sample images of MTS-mCherry and MTS-Fluc(WT)-mCherry in Fig S2, S3 and referenced them in the primary manuscript (lines 174-182, 643-646, 689-694).

R2.3: Related to the above point, the DUMP cells (+ and -) have lost membrane potential. This brings in other confounding variables associated with mitophagy potentially. So this may be an amplified fission expt rather than normal rates of fission. And how is the luciferase protein impacting membrane potential? Even when it is supposedly not misfolded?

In our studies, we grow all yeast cells in glucose-rich media (YPD), where mitophagy rarely occurs. It is known that in yeast mitophagy is most frequently observed when cells are grown in the presence of non-fermentable carbons (Fukuda & Kanki, 2018; Kanki et al., 2009; Kanki & Klionsky, 2008). Our previous work (Ruan et al., 2020) showed that DUMP-containing mitochondria are stable and there was no evidence that DUMP induces mitophagy. We also showed in previous work that DUMP formation reduces membrane potential likely by sequestration of TCA cycle enzymes. We acknowledge that detailed mechanism by which DUMP impairs mitochondrial fitness remains obscure, but we do not feel that is a critical issue for the present study.

R2.4: Diffusive motion suggests that mitochondria move randomly in the cell, but they are often moving along microtubules by motors or being pushed by actin. This is not directly incorporated in the diffusion parameter with any vector. But is this achieved by using a 2D plane to limit conformational freedom? And how does this limit the model?

In yeast microtubules do not play any role in mitochondrial movement whereas actin cables facilitate mitochondrial transport from the mother to daughter cell. We observed that mitochondria primarily exhibit directed motion during brief periods in the cell cycle when they transported through the bud neck into the bud (Fig 1G-H). Outside of these stages, discerning between directed and diffusive motion becomes challenging, as the movements appear more random in nature. Our choice to model this predominantly as diffusive motion in a 2D plane was an effort to encapsulate the predominant behavior of mitochondria along the cell cortex during periods prior to transport and passage through bud neck. We have mentioned this in lines 808-814.

R2.5: It is not clear to me whether the DUMP mitos that preferentially divide/separate are more prone to fuse/interact with adjacent mitochondria in the model. If so, they are essentially stuck in this local cycle of fission and fusion? Is that the idea? And if so, what would be the proposed mechanism in a cellular environment that promotes this cycling?

Once mitochondria undergo fission, the decision for the resulting two mitochondria to undergo fusion is not based on their history. In other words, there is not a cyclical mechanism whereby DUMP mitochondria are predisposed to continuously fission and fuse. Instead, their interactions

are governed by the general fusion parameters set within the model. We have further clarified this in the updated model description to eliminate any ambiguities (lines 970-979).

R2.6: In the data for figure 5, it would be better to have a co-localization measure (Pearson Coefficient or something similar) to assess Dnm1 interactions with DUMP. The number of punctae (N) is less informative since there are many other punctae on the mitochondria. This is done later, but it would be a good practice when showing co-localization in cells.

We appreciate the reviewer's suggestion to use the Pearson Coefficient (PCC) for assessing co-localization. However, using the PCC poses challenges in our specific system, particularly because it cannot differentiate between DUMP vs non-DUMP regions of DUMP+ mitochondria without manually segmenting specific regions of the 3D image. Given that mitochondria do not conform to rectilinear shapes, this manual segmentation might introduce inaccuracies. It is also pertinent to note that Dnm1 is not exclusively localized around DUMP; it can be found elsewhere on the mitochondria, albeit with differing surface densities.

While PCC is a valuable tool, we believe our chosen method effectively captures the co-localization information we aim to convey and have further elaborated on our rationale in the methods section (lines 866-888).

R2.7: The deletion of Mdv1 and Caf4 are not affecting Dnm1 localization to DUMP sites. The authors show ΔFis1 in the supplemental data, but there appear to be a number of additional Dnm1 punctae that form at locations other than mitochondria. This complicates the interpretation and the co-localization is less clear in the image presented. Better quantitative assessment of the co-localization would help. Additionally, GFP has been shown to promote assembly in the mammalian homolog of Dnm1p, Drp1. A similar phenomenon might be true in these expts, which is why so many punctae are observed.

We acknowledge the reviewer's observations regarding the presence of Dnm1 punctae that are not bound to mitochondria. It is not unexpected that by deleting more than two components of fission machinery, a proportion of Dnm1 disassociates from the mitochondria. While the dissociated Dnm1 could still cluster, these cytosolic punctae are irrelevant to mitochondrial fission. Our imaging also showed that Dnm1 does not always present in a punctate manner, but can also appear in diffuse, which complicates quantification (Fig S10). However, our primary objective in presenting these images was to illustrate that the loss or impairment of specific fission machinery components, responsible for anchoring Dnm1, leads to Dnm1 dissociation from the mitochondria. We have added a quantification of the GFP-Dnm1 intensity on mitochondria vs. the cytoplasm to demonstrate this dissociation (Fig S11, lines 880-888).

R2.8: I don't understand Fig S9. This is supposed to demonstrate the rates of fission and fusion, but I don't see any difference between the averages in the different strains (WT vs deletions). It looks like there are very few events every 10 s. Is there a difference if a longer timepoint is considered? I may just not understand the graph tbh.

Figure S13 shows only a control to demonstrate that expression of mitoFluc does not cause any change in fission/fusion rates that could have contributed to altering probability of DUMP inheritance. There are no differences in fission/fusion rates at longer time scales.

R2.9: Perhaps the most interesting results is that you need Mgm1 to get matrix mixing that facilitates the consolidation of the DUMPs. Is the next step to identify the coordination of fission and fusion to isolate these damaged regions? Discussion on the extent to which fusion contributes to this process would be warranted. The discussion focused solely on fission.

Indeed, the role of Mgm1 in facilitating matrix mixing, which subsequently aids in the consolidation of DUMPs, stands out as one of our most intriguing findings. Reviewer's insights on the potential coordination of fission and fusion in isolating these damaged regions are noteworthy. We have therefore broadened the scope of our discussion to incorporate the role of fusion more comprehensively (lines 613-627).

pg 29 - The authors refer to Mdv1 as a "membrane-bound protein", but I believe that this is not true. It associates with the membrane through interactions with Fis1. Mdv1 does not have a membrane binding region to my knowledge.

The authors state that "deletion of CAF4, a gene encoding an Mdv1 paralog [69] that does not form puncta (Fig S7A, B)", but this is actually Fig S6, H-I)

We are grateful to the reviewer for pointing out these inaccuracies. Mdv1 indeed does not directly bind membrane but associates with the membrane via interactions with Fis1. We have also rectified the incorrect figure reference.

Fukuda, T., & Kanki, T. (2018). Mechanisms and physiological roles of mitophagy in yeast. *Molecules and Cells*, *41*(1), 35–44. https://doi.org/10.14348/molcells.2018.2214

Kanki, T., & Klionsky, D. J. (2008). Mitophagy in yeast occurs through a selective mechanism. *Journal of Biological Chemistry*, *283*(47), 32386–32393. https://doi.org/10.1074/jbc.M802403200

Kanki, T., Wang, K., Baba, M., Bartholomew, C. R., Lynch-Day, M. A., Du, Z., Geng, J., Mao, K., Yang, Z., Yen, W. L., & Klionsky, D. J. (2009). A genomic screen for yeast mutants defective in selective mitochondria autophagy. *Molecular Biology of the Cell*, *20*(22). https://doi.org/10.1091/mbc.E09-03-0225

Ruan, L., McNamara, J. T., Zhang, X., Chang, A. C.-C., Zhu, J., Dong, Y., Sun, G., Peterson, A., Na, C. H., & Li, R. (2020). Solid-phase inclusion as a mechanism for regulating unfolded proteins in the mitochondrial matrix. *Science Advances*, *6*(32), eabc7288.
